# Elderly Patients with Idiopathic Pulmonary Hypertension: Clinical Characteristics, Survival, and Risk Stratification in a Single-Center Prospective Registry

**DOI:** 10.3390/life14020259

**Published:** 2024-02-16

**Authors:** Natalia Goncharova, Kirill Lapshin, Aelita Berezina, Maria Simakova, Alexandr Marichev, Irina Zlobina, Narek Marukyan, Kirill Malikov, Alexandra Aseeva, Vadim Zaitsev, Olga Moiseeva

**Affiliations:** Almazov National Medical Research Centre, Ministry of Health of Russia, Saint Petersburg 197341, Russiaberezina_av@almazovcentre.ru (A.B.); marichev_ao@almazovcentre.ru (A.M.); zlobina_is@almazovcentre.ru (I.Z.); marukyan_nv@almazovcentre.ru (N.M.); malikov_kn@almazovcentre.ru (K.M.); aseeva_as@almazovcentre.ru (A.A.); zaytsev_vv@almazovcentre.ru (V.Z.); moiseeva_om@almazovcentre.ru (O.M.)

**Keywords:** idiopathic pulmonary arterial hypertension, comorbidities, survival, risk stratification

## Abstract

Introduction: The predictive value of the risk stratification scales in elderly patients with IPAH might differ from that in younger patients. It is unknown whether young and older IPAH patients have the same survival dependence on PAH-specific therapy numbers. The aim of this study was to evaluate the prognostic relevance of risk stratification scales and PAH medication numbers in elderly IPAH patients in comparison with young IPAH patients. Materials and methods: A total of 119 patients from a prospective single-center PAH registry were divided into group I < 60 years old (n = 89) and group II ≥ 60 years old (n = 30). ESC/ERS, REVEAL, and REVEAL 2.0 risk stratification scores were assessed at baseline, as well as H2FpEF score and survival at follow-up. Results: During a mean follow-up period of 2.9 years (1.63; 6.0), 42 (35.3%) patients died; at 1, 2, 3, 5, 7, and 10 years, survival was 95%, 88.6%, 78.5%, 61.7%, 48.5%, and 33.7%, respectively. No survival differences were observed between the two groups, despite the use of monotherapy in the elderly patients. The best predictive REVEAL value in elderly patients (IPAH patients ≥ 60 years) was AUC 0.73 (0.56–0.91), *p* = 0.03; and in patients with LHD comorbidities in the entire cohort, it was AUC 0.73 (0.59–0.87), *p* < 0.009. Factors independently associated with death in the entire cohort were CKD (*p* = 0.01, HR 0.2), the right-to-left ventricle dimension ratio (*p* = 0.0047, HR 5.97), and NT-proBNP > 1400 pg/mL (*p* = 0.008, HR 3.18). Conclusion: Risk stratification in the elderly IPAH patients requires a fundamentally different approach than that of younger patients, taking into account the initial limitations in physical performance and comorbidities that interfere with current assessment scores. The REVEAL score reliably stratifies patients at any age and LHD comorbidities. The initial monotherapy seems to be reasonable in patients over 60 years. Selection tools for initial combination PAH therapy in older IPAH patients with comorbidities need to be validated in prospective observational studies.

## 1. Introduction

IPAH is a devastating disease with a dismal prognosis. An adequate assessment of IPAH’s severity and prognosis is essential for an optimal treatment strategy choice [1]. The ESC/ERS 2015, REVEAL (Registry Risk Score for Pulmonary Arterial Hypertension), and REVEAL 2.0 scales are the most used in clinical practice for the severity and prognosis assessment in patients with IPAH. The ESC/ERS 2015 scale was created by PAH experts based on collective opinion regarding the results of studies and observations in patients with IPAH at the time of diagnosis. The ESC/ERS scale considers clinical (syncope, signs of the right heart failure, functional class (WHO)), hemodynamic parameters (right atrial pressure, cardiac index), an oximetry study (mixed venous oxygen saturation), echocardiography or cardiac magnetic resonance imaging of the right heart’s size and functional assessment, serum natriuretic peptides concentration, and exercise intolerance assessment (six-minute-walk test or cardiorespiratory testing) [2,3,4,5]. All these parameters could be modified with the treatment. The REVEAL scale formation was based on the results of a multivariate analysis of the REVEAL registry (The Registry to Evaluate Early and Long-term PAH Disease Management), which included predominantly prevalent Caucasian (72.5%) PAH group I patients in the USA [6]. The advantage of the REVEAL and REVEAL 2.0 scales was the inclusion of unmodifiable parameters, like PAH etiology, age, gender, and the fact of hospitalization due to PAH [7]. Comorbidities were not included in any of the existing risk scales, with the exception of the estimated glomerular filtration rate (eGFR), which was a part of the REVEAL and REVEAL 2.0 scales.

The aggressive nature of IPAH with an unacceptable mortality rate has led to the idea of an upfront combination of PAH therapy at initiation, even in low-risk IPAH patients with negative vasoreactive testing [4,5]. The dramatic shift of the mean age in IPAH patients towards the 6th decade of life in the current registries is an important issue for the treatment options [8]. The diagnosis of IPAH in older populations raises concern as these patients usually have a spectrum of comorbidities that influence hemodynamics and clinical presentation [9,10] and could modify treatment effect [11]. At present, there is no countable score or tool for the evaluation of the left heart or lung disease significance in patients with precapillary pulmonary hypertension [12]. M. Hoeper et al. (2018) suggested a revised treatment strategy taking into account age and comorbidity phenotype, referring to initial combination PAH therapy for young IPAH patients without comorbidities [13]. The ESC/ERS 2022 guidelines on PAH management have supported the concept of initial monotherapy in IPAH patients with a left heart or lung disease despite their risky status [5]. Nevertheless, it is not well-defined whether a treatment approach with initial monotherapy in elderly IPAH patients with comorbidities could influence survival.

This study aimed to evaluate the prognostic relevance of risk stratification scales and PAH medication numbers in elderly IPAH patients in comparison with young IPAH patients.

## 2. Methods

### 2.1. Data Collection

The Almazov National Medical Research Center initiated a prospective PAH program for adult patients in January 2006 in the Northwest region of Russia (*additional information in Supplementary Materials). The inclusion criteria into the registry for patients with precapillary pulmonary hypertension were the following: a mean pulmonary arterial pressure (mean PAP) ≥ 25 mmHg, pulmonary capillary wedge pressure (PCWP) < 15 mmHg, and pulmonary vascular resistance (PVR) ≥ 3 Wood units.

**Figure 1:**
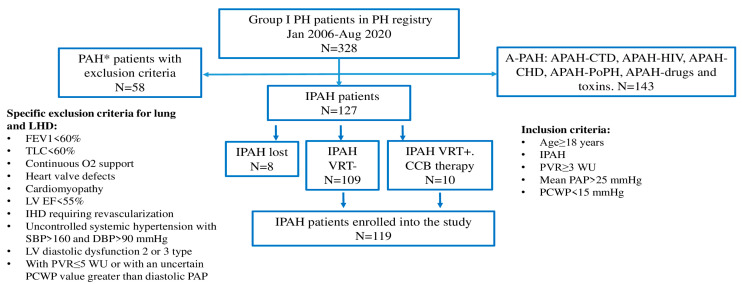
Inclusion to the study. * patients with precapillary pulmonary hypertension (PVR ≥ 3 WU, mPAP ≥ 25 mmHg, PCWP < 15 mmHg) and with exclusion criteria presence; A-PAH, pulmonary arterial hypertension associated with other diseases and conditions; APAH-CTD, pulmonary arterial hypertension associated with connective tissue disease; APAH-HIV, pulmonary arterial hypertension associated with human immunodeficiency virus; APAH-CHD, pulmonary arterial hypertension associated with congenital heart defects (patients with corrected CHD were enrolled in the registry); APAH-PoPH, pulmonary arterial hypertension associated with portopulmonary hypertension; DBP, diastolic blood pressure; IHD, ischemic heart disease IPAH, idiopathic pulmonary arterial hypertension; FEV1, forced expiratory volume in one second; LHD, left heart disease; LV EF, left ventricular ejection fraction; PAP, pulmonary arterial pressure; PCWP, pulmonary capillary wedge pressure; PVR, pulmonary vascular resistance; TLC, total lung capacity; VRT, vasoreactive testing.

The exclusion criteria for the study were the following: other PAH etiologies than IPAH, moderate to severe lung disease, severe left heart disease, established malignancies, uncontrolled diabetes mellitus, uncontrolled thyroid disease, mental disorders, and end-stage kidney dysfunction (Figure 1). A left heart disease (LHD) phenotype was defined as having more than 3 comorbidities: systemic hypertension, ischemic heart disease, diastolic dysfunction by echocardiography, atrial fibrillation, or obesity (IMT > 30 kg/m^2^).

Demographics, symptoms, comorbidity, 6 min walk test (6MWT) distance and cardiopulmonary exercise test (CPET), echocardiographic and RHC parameters, lung function test including diffusion capacity for carbon monoxide (DLCO), and laboratory variables (hemoglobin, creatinine with eGFR, total bilirubin, uric acid, N-terminal pro-brain-type natriuretic peptide [NT-proBNP]) were collected in a period of 1 month, when the first right heart catheterization (RHC) at the PH referral center was performed. The number of PAH medications was evaluated 3 months after the IPAH diagnosis confirmation and at the end of the follow-up. The estimated GFR was calculated according to the CKD-EPI equation.

Baseline risk stratification was performed using 2015 ESC/ERS risk stratification, the REVEAL (Registry to Evaluate Early and Long-Term Disease Management), and the REVEAL 2.0 score [https://www.pahinitiative.com/hcp/risk–assessment/calculators (URL accessed on 12 February 2024)] with the parameters obtained in 1 month, when the first right heart catheterization (RHC) at the PH referral center was performed. The probability of heart failure with preserved ejection fraction (HFpEF) was assessed using the heart failure with preserved ejection fraction (H2FpEF) score (Heavy-Hypertensive (2 or more antihypertensive medicines)—paroxysmal or persistent Atrial Fibrillation—Elder (age > 60 years)—Filling pressure (Doppler echocardiographic E/e_(l)_ ratio > 9)—Doppler echocardiographic estimated systolic pulmonary arterial pressure > 35 mmHg) [14]. H2FpEF score was calculated without taking into account the presence of pulmonary hypertension as all patients were with IPAH. H2FpEF was chosen for its high sensitivity (0.70) and specificity (0.91) in the PH group II prediction in patients with elevated estimated systolic pulmonary artery pressure [15].

The number of PAH medications was assessed at the time of diagnosis and the end of follow-up.

The start date of follow-up was the date of hospitalization for the first RHC, and the last date of follow-up was the date of death, last patient visit, or telephone contact within three months. Four incident patients were enrolled into the study from June 2020 to September 2020; then, the enrollment was closed in order to have at least 6 months of follow-up. Patients had regular follow-up with the first check-up in 3 months after PAH therapy initiation, followed by regular 6-month evaluations. Visits were performed at the PH referral center or at regional PH centers and general cardiology outpatient clinics. All regional PH centers are supervised by the Northwest referral PH center. The date and cause of death were obtained from medical records, local physicians, or death certificates provided by relatives (**additional information in Supplementary Materials). The follow-up analysis was limited to February 2021.

By the legislation of the Russian Federation and the Local Acts of the Almazov National Medical Research Center, a statistical analysis of registries does not require a specific permission of an Ethics Committee. The study reflects daily clinical practice within the guidelines for the management of patients with PAH [2,3,5]. Identifiable patient information was not presented in the study.

### 2.2. Study Population

The study population comprised 119 (≥18 years old) Caucasian IPAH patients (Figure 1). All patients were divided according to age of IPAH diagnosis into two groups: group I < 60 years old (n = 89); group II ≥ 60 years old (n = 30). The entire cohort of IPAH patients was divided into sub-groups based on H2FpEF score: H2FpEF score ≤ 1, H2FpEF score ≥ 2. Lung function tests were performed in 102 patients, NT-proBNP in 112 patients, 6MWT in 118 patients, and CPET in 76 patients.

### 2.3. Statistical Analysis

Demography, clinical data including comorbidity, PAH functional class (FC) (WHO), PAH drug number, hemodynamic data, laboratory and lung function test parameters, echocardiography variables, and exercise performance parameters, obtained at baseline, were compared in the young and elderly groups as well as in the H2FpEF score groups. Numerical parameters with a normal distribution were presented as mean ± standard deviation (M ± SD), and numerical parameters with an abnormal distribution were presented as median and interquartile range (IQR; M ± 25%, 75%). Categorical variables were presented as absolute numbers and percentages and compared using Fisher exact or Pearson M-L Chi-square tests, as appropriate. Mean values in groups were compared using the Mann–Whitney U-test and Wilcoxon signed-rank test, as appropriate. Survival analyses were performed using Kaplan–Meier curves and log-rank test to compare survival distribution between young and elderly patients as well as for the patients with or without left heart disease (LHD) comorbidities. The discrimination power of the different risk stratification scoring systems was evaluated using the area under the receiver operating characteristic (ROC) curve (AUC). A statistically significant difference was determined as a two-tailed *p* < 0.05. The statistical analyses of the data were carried out using Statistica for Windows, version 10.0 (StatSoft: Tulsa, Okla, USA).

## 3. Results

### 3.1. Study Population, Comorbidity, and Exercise Tolerance

The mean age was 45.19 ± 16.18 years [min-max: 18; 76], with a male-to-female ratio of 1:5.4 in the entire cohort (n = 119). The mean time from symptoms to the first RHC was 1.9 (0.9; 3.9) years, and the mean follow-up was 2.98 (1.6; 6.1) years in the entire study population. Patients with comorbidities were more prevalent in the elderly group compared with patients in the young group (Table 1).

Patients with systemic hypertension and COPD had shorter 6MWT distances (280 ± 125 vs. 343 ± 129 m, *p* = 0.002; and 227 ± 138 vs. 329 ± 126 m, *p* = 0.007) when compared with other patients in the entire IPAH population. Patients with permanent atrial fibrillation or atrial flutter IHD had a shorter 6MWT distance (235 ± 127 vs. 329 ± 128 m, *p* = 0.01; and 208 ± 121 vs. 331 ± 126 m, *p* = 0.001) when compared with other patients in the entire IPAH cohort. No significant correlations were revealed between the 6MWT distance and diabetes mellitus, presence of chronic kidney disease, and smoking in the entire population. A negative correlation (ρ = −0.16, *p* = 0.07) was observed between BMI and the 6MWT distance.

A negative correlation was found between the age and SatO_2_ (ρ = −0.39, *p* < 0.001) and with the Tiffno index (ρ = −0.26, *p* < 0.01). The presence of COPD was associated with a tendency for a lower SatO_2_ (92.4 ± 4.3 vs. 94.6 ± 3.7%, *t*-test, *p* = 0.06) in the entire cohort of patients. The presence of obesity and smoking history were associated with a lower SatO_2_ (*p* = 0.01; *p* = 0.01, respectively).

Patients with III-IV FC (WHO) were more prevalent in the elderly group in comparison with the young patients. The 6MWT distance was significantly reduced in the elderly patients. But the peak O_2_ consumption (VO_2_), as well as oxygen pulse (VO_2/_HR) at the aerobic threshold in an absolute value, were comparable between the two groups. To exclude age-dependent differences in the parameters above, the percentages from the predicted values were analyzed. The percentage of peak VO_2_ predicted, percentage of aerobic threshold predicted, and percentage of VO_2_/HR predicted were significantly lower in younger patients in comparison with the elderly group. Minute ventilation per unit carbon dioxide production (VE/VCO_2_) on peak exercise did not differ between groups, whereas VE/VCO_2_ on aerobic threshold was lower in the younger patients compared with the elderly patients. Breathing reserve was significantly lower in the elderly patients (Table 1).

### 3.2. Echocardiography Data

Left atrium (LA) volume index and LV end-diastolic dimension were larger in the elderly group when compared with the young patients. No significant differences in the sizes of the right heart and function were registered between the two groups. A trend towards a higher right-to-left ventricular end-diastolic dimension ratio (RV/LV ratio) was found in the young patients in comparison with the elderly patients (Table 2). No differences were registered in stroke volume derived from echocardiography and the LV ejection fraction between the two groups.

A lower median LA volume index was found in the deceased patients: 21.5 [18.0; 26.0] vs. 25.5 mL/m^2^ [22.0; 34.1], *p* = 0.001, and in patients with cardiac index less than 2.0 l/min/m^2^: 24.0 [19.0; 28.0] vs. 27.0; [23.0; 35.0] ml/m^2^, *p* = 0.01. A smaller mean LA diameter was registered in patients with syncope (34.4 ± 6.7 vs. 38.2 ± 7.3 mm, *p* = 0.003).

In patients with PAH III-IV FC (WHO), the LV end-diastolic dimension (62.7 ± 19.4 vs. 80.7 ± 23.3 mm, *p* < 0.001) and LV stroke volume were smaller than in the I-II FC patients (41.3 ± 13.5 vs. 54.1 ± 14.4 mL, *p* < 0.0001). A smaller LV end-diastolic dimension was associated with a cardiac index less than 2.0 l/min/m^2^ (*p* < 0.001) and NT-proBNP > 1400 pg/mL (*p* < 0.0001).

### 3.3. Hemodynamic Data

Patients in the young group exhibited a significantly lower mean blood pressure, higher heart rate, and mean PAP in comparison with the elderly patients (Table 3).

Patients with the LHD phenotype had a significantly higher mean blood pressure (95.2 ± 13.4 vs. 85.3 ± 14.8 mm Hg, *p* = 0.005) and lower PVR (PVR: 11.8 ± 4.3 vs. 15.2 ± 7.9 WU, *p* = 0.0087) when compared with patients without LHD.

Obesity was associated with a lower PVR index (6.1 ± 2.5 vs. 9.15 ± 4.9 WU/m^2^; *p* < 0.001), which was supported by the inverse correlation between the body mass index and PVR (ρ = −0.26; *p* = 0.003).

### 3.4. Laboratory Data

Estimated GFR was markedly decreased in elderly patients in comparison with young patients, whereas no significant difference was registered in hemoglobin, NT-proBNP, creatinine, bilirubin, and uric acid concentrations between the two groups (Table 1). Estimated GFR was lower in patients with systemic hypertension in comparison with patients without systemic hypertension (72.6 ± 22.9 vs. 83.2 ± 23.8 mL/min/1.73 m^2^, *p* = 0.01). A positive correlation was detected between the age and uric acid (ρ = 0.30, *p* = 0.006), creatinine (ρ = 0.22, *p* = 0.017), and NT-proBNP levels in serum (ρ = 0.17, *p* = 0.06). The inverse correlation between the eGFR and NT-proBNP levels in serum was just about the statistical value of confidence (ρ = −0.18, *p* = 0.05) in the entire cohort of patients.

### 3.5. Risk Stratification

#### 3.5.1. Entire IPAH Group (n = 119)

**Figure 2:**
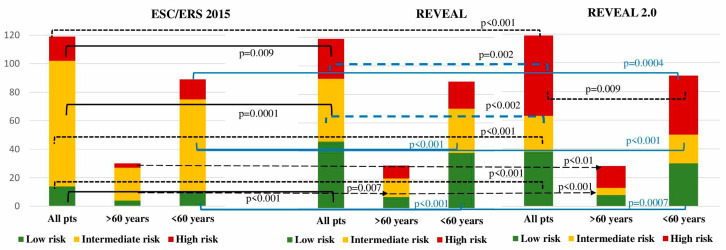
Number of IPAH patients according to the different risk stratification scales (significance of differences between risk groups is indicated only for *p* < 0.05).

Patients were stratified according to the ESC/ERS 2015, REVEAL, and REVEAL 2.0 risk stratification scales. Considerable differences were registered in between the risk stratification scales when applied to the entire cohort of patients. Significantly more high-risk patients were observed using the REVEAL 2.0 compared to the ESC/ERS risk scale (47 vs. 14.3%, *p* < 0.001), while with the REVEAL scale, 23.5% patients were appraised as a being high-risk (*p* = 0.009 compared to the ESC/ERS). Similar inconsistencies were observed regarding the low-risk and intermediate-risk numbers of patients between ESC/ERS and REVEAL (Figure 2). Intermediate-risk patients in the entire IPAH population were the predominant group according to the ESC/ERS scale in comparison with the REVEAL and REVEAL 2.0 scales (73.1 vs. 36.9 vs. 21%; *p* < 0.001; *p* < 0.0001, respectively). The REVEAL and REVEAL 2.0 scales corresponded with each other in the low-risk patient stratification (39.5 vs. 32%, *p* = 0.14), whereas high-risk patients were more prevalent with the REVEAL 2.0 score (47 vs. 25.3%, *p* = 0.002). More patients with intermediate risk were stratified with a REVEAL score in comparison with REVEAL 2.0 (36.9 vs. 21%, *p* = 0.01) (Table 4).

#### 3.5.2. Young Group (under 60 Years)

A significant difference in the low-risk patient numbers was found through a comparison between the scales of ESC/ERS, REVEAL (12.3 vs. 43.8%, *p* < 0.001), and REVEAL 2.0 (12.3 vs. 34.8%, *p* = 0.0007). According to the ESC/ERS, the majority of patients were intermediate-risk in comparing with REVEAL (71.9 vs. 34.8%, *p* < 0.001) and REVEAL 2.0 scales (71.9 vs. 24.7%, *p* < 0.001). The lowest proportion of high-risk patients was stratified with ESC/ERS in comparison with REVEAL (15.7 vs. 21.3%, *p* = 0.4) and REVEAL 2.0 (15.7 vs. 40.4%, *p* = 0.0004). No differences were registered between REVEAL and REVEAL 2.0 in the stratification of low- (*p* = 0.2) and intermediate-risk patients (*p* = 0.18). A considerably elevated proportion of high-risk patients was stratified according to REVEAL 2.0 compared with REVEAL (40.4 vs. 21.3%, *p* = 0.009) (Table 4, Figure 2).

#### 3.5.3. Elderly Group (over 60 Years)

No differences in low-risk patients were observed between ESC/ERS, REVEAL (10 vs. 26.6%, *p* = 0.2), and REVEAL 2.0 (10 vs. 23.3%, *p* = 0.2) systems. The intermediate risk was allocated to the majority of patients with the ESC/ERS scale in comparison with REVEAL (80 vs. 43.3%, *p* = 0.007) and REVEAL 2.0 (80 vs. 10%, *p* < 0.001). A high risk was attributed to the minority of patients according to the ESC/ERS scale compared to REVEAL (10 vs. 30%, *p* = 0.1) and REVEAL 2.0 (10 vs. 66.6%, *p* < 0.001). The numbers of the low-risk group did not differ between REVEAL and REVEAL 2.0 scores (*p* = 1), whereas a significant difference was registered between the REVEAL and REVEAL 2.0 scores in the intermediate- (*p* = 0.007) and high-risk (*p* = 0.009) groups.

No differences in risk were observed between two groups using ESC/ERS and REVEAL. Only REVEAL 2.0 exhibited an increased percentage of high-risk patients in the elderly group when compared with the young group of patients (60.6 vs. 40.4%, *p* = 0.02) (Table 4, Figure 2).

### 3.6. H2FpEF-Score

H2FpEF-score ≤ 1 was registered in 74 patients (62.2%), and H2FpEF score ≥ 2 in 45 patients (37.8%) in the entire cohort. Patients with H2FpEF score ≥ 2 had significantly lower 6MWT distance, eGFR, and SatO_2_, whereas mean BP and right atrial pressure (RAP) were significantly elevated in comparison with patients with H2FpEF score ≤ 1. H2FpEF score ≥ 2 was more prevalent in elderly patients in comparison with the younger group (51 vs. 9.5%, *p* = 0.0001). No significant differences in PCWP, cardiac index, and PVR were observed between the H2FpEF scores in the entire IPAH cohort. We did not find any significant differences between the NT-proBNP and the number of PAH medications prescribed to the patients by the H2FpEF score in the entire cohort of patients. Loop diuretics were used more often in patients with H2FpEF score ≥ 2 in comparison with H2FpEF score ≤ 1 (Table 5). In the elderly group, the number of patients with H2FpEF score ≤ 1 was very limited (n = 7, 23.3%), failing to demonstrate the true differences with patients having H2FpEF score ≥ 2.

### 3.7. Survival Analysis

**Figure 3:**
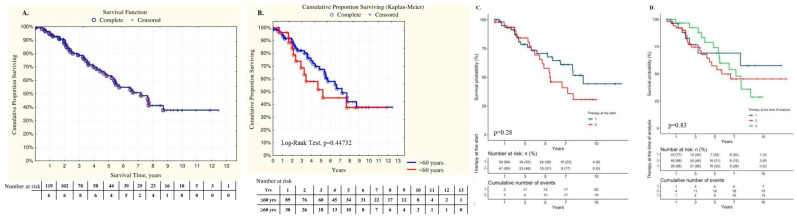
Kaplan–Meier survival curves: (**A**). Survival in the entire IPAH patients cohort; (**B**). Survival in the young group and elderly group of patients; (**C**). Survival in the entire IPAH cohort of patients (n = 119) through the initial PAH therapy; (**D**). Survival in the entire IPAH cohort with PAH therapy at the time of analyses.

In the entire cohort (n = 116), all-cause mortality was 35.3% (n = 42). The main cause of death was PAH progression in 76% (n = 32), sudden death was reported in seven cases (16.6%), pneumonia in one patient (2.4%), pneumothorax during RHC in one patient (2.4%), and one case (2.4%) of severe gastric bleeding. For the entire cohort, the Kaplan–Meier estimated survival rates for 1, 2, 3, 5, 7, and 10 years after diagnosis were 95%, 88.6%, 78.5%, 61.7%, 48.5%, and 33.7%, respectively. Mortality rates did not differ between the two groups (Table 4), as well as survival rates (Figure 3). No difference in survival was registered in patients with (n = 49) and without LHD comorbidities (n = 70) (Log-rank test, *p* = 0.65). No significant differences in survival were observed in the entire IPAH cohort in different H2FpEF-score groups (Log-rank test, *p* = 0.2).

**Figure 4:**
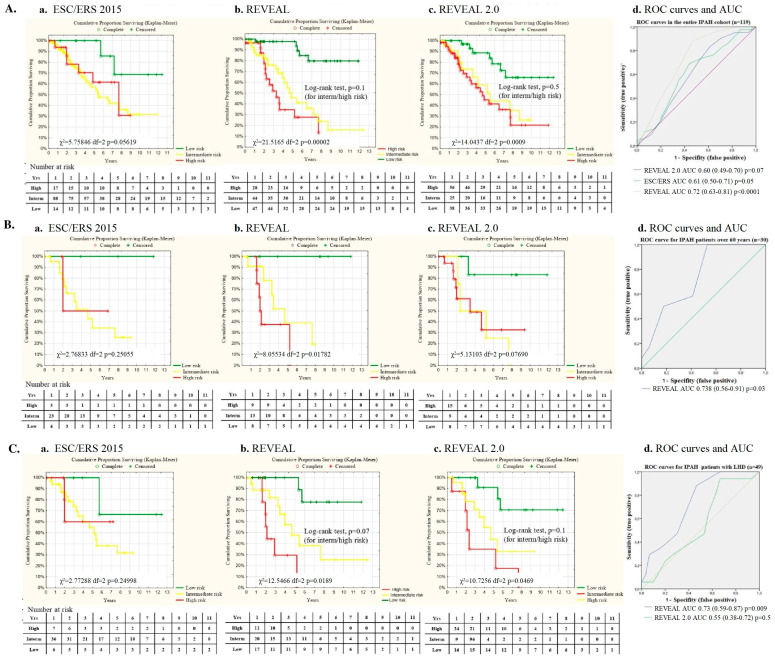
Survival according to the risk stratification and ROC curves and the associated area under the curve (AUC) for the risk stratification scales: (**A**). Survival in the entire IPAH population (n = 119) **a.** according to the ESC/ERS scale; **b.** according to the REVEAL scale; **c.** according to the REVEAL 2.0 scale; **d.** Receiver operating characteristics (ROC curves) for the ESC/ERS, REVEAL and REVEAL 2.0 scales in entire IPAH population; (**B**). Survival in the elderly IPAH patients (n=30) **a.** according to the ESC/ERS scale; **b.** according to the REVEAL scale; **c.** according to the REVEAL 2.0 scale; **d.** Receiver operating characteristics (ROC curves) for REVEAL scale in the elderly IPAH patients (n = 30); (**C**). Survival in the IPAH pts with LHD comorbidities (n = 49) **a.** according to the ESC/ERS scale; **b.** according to the REVEAL scale; **c.** according to the REVEAL 2.0 scale; **d.** Receiver operating characteristics (ROC curves) for REVEAL scale in the IPAH pts with LHD comorbidities (n = 49).

The REVEAL score exhibited the strongest predictive value between low-, intermediate-, and high-risk patient survival when comparing the REVEAL 2.0 and ESC/ERS scores in the entire IPAH patients’ cohort (Figure 4A,D). Nevertheless, no significant discrimination was observed in survival in IPAH patients with intermediate and high risk at baseline.

In the elderly group, the best predictive value for survival was observed when using the REVEAL risk score, whereas survival prediction did not reach statistical significance when using the REVEAL 2.0 and ESC/ERS scores (Figure 4B,D).

The REVEAL score reliably predicted survival in patients with LHD comorbidities (n = 49), whereas the predictive value for the REVEAL 2.0 score was statistically insignificant, as well as the ESC/ERS score (χ^2^ = 2.7, *p* = 0.2) (Figure 4C,D).

### 3.8. PAH-Specific Therapy

Almost all patients had been receiving PAH-specific therapy within the first 6 months since the diagnosis of IPAH was confirmed. The majority of patients started with monotherapy 59.6% (n = 71), 39.4% (n = 47) with double-combination therapy, and only 1 patient (0.84%) with triple-combination PAH therapy. Ten patients had a positive vasoreactive test and were receiving calcium channel blocker (CCB) therapy at baseline. Seven patients were receiving CCB therapy at the end of follow-up. Patients with positive vasoreactive test and CCB therapy were in a group < 60 years.

**Figure 5:**
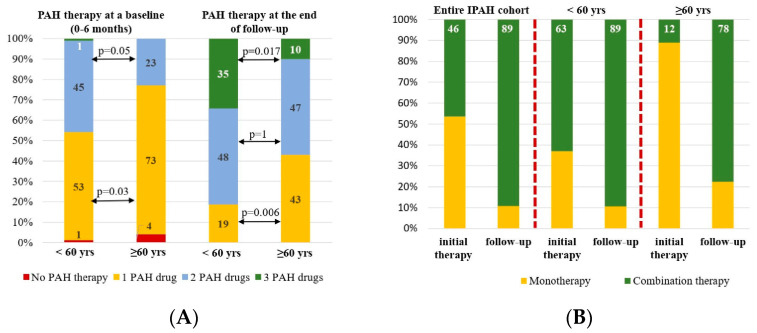
PAH therapy in the entire IPAH cohort, young group (under 60 years), elderly group (over 60 years): (**A**). PAH drug numbers at baseline and follow-up (**B**). PAH therapy in high-risk patients with IPAH according to REVEAL risk stratification.

The elderly patients were significantly less likely to receive initial combination therapy in comparison with the young patients (χ^2^ = 4.8, *p* = 0.028). Sildenafil was the drug most used in mono- and combination PAH therapies. At the time of the analysis, the difference in the number of PAH drugs was even more pronounced than at baseline with more numbers for the young patients compared to the elderly group of patients (χ^2^ = 9.49, *p* = 0.008). The observed difference in the PAH drug number was particularly evident in the young patients for the triple-combination therapy (χ^2^ = 5.82; *p* = 0.016). No statistical differences were observed in the double-combination PAH therapy between the two groups at the time of analysis (48.3 vs. 46.6% in elderly group, *p* = 0.1). Nearly half (43.3%) of the patients in the elderly group received monotherapy in comparison with only 19.1% of patients in the young group (χ^2^ = 6.98; *p* = 0.008) (Table 4, Figure 5).

Twenty-eight patients were stratified as high-risk according to the REVEAL score in the entire population, 19 patients in the young group, and 9 patients in the elderly group of patients. Initial combination therapy had been started in 13 patients (46.4%) in the entire group: among those, 12 patients (63%) were in the young group and only 1 patient (11.1%) in the elderly group (*p* = 0.015). During the follow-up, the number of patients receiving combination PAH therapy increased up to 24 patients (89.3%) predominantly among the elderly patients (n = 7, 77.7%, *p* = 0.01) and fewer among the young patients (n = 17, 89.5%, *p* = 0.2) (Figure 5).

No significant differences in survival were registered in patients with mono- or combination PAH-specific therapies at baseline and at the time of analysis in the entire IPAH cohort (Figure 3C,D). The same observation was registered in the elderly patients (*p* = 0.6) as well as in the IPAH patients with LHD (*p* = 0.8).

Seventy-two patients were stratified as intermediate and high-risk according to the REVEAL score in the entire IPAH cohort. No significant correlations were registered between the initial number of PAH drugs and survival for the intermediate (*p* = 0.9) and high-risk group (*p* = 0.2). Inhaled iloprost was used as a third PAH drug only in 22 patients, predominantly in the young group of patients (n = 20).

### 3.9. Factors Associated with Death

Demographics, symptoms, comorbidity, H2FpEF score, 6MWT distance, CPET parameters, echocardiographic and RHC parameters, lung function test including DLCO, and laboratory variables were analyzed in association with death in the entire cohort of IPAH patients using univariate Cox regression analysis with subsequent selection of factors that had a statistically significant association with mortality, that had the highest level of risk ratio, and that were well established according to previous studies on IPAH patients [5,15] (Table 6, online Supplements). Chronic kidney disease presence, the RV/LV ratio, and NT-proBNP >1400 pg/mL were independently associated with death in the entire cohort of IPAH patients (Table 7). Cardiac index and mixed venous oxygen saturation were eliminated from the analyses due to their tight interrelationship with each other and NT-proBNP.

## 4. Discussion

The main findings of our study are the evidence of a high discrepancy in risk stratification between ESC/ERS 2015, REVEAL, and REVEAL 2.0 scores and the insufficient relevance of the scales for patients over 60 years and patients with comorbidities. The absence of differences in survival between the young and elderly groups of IPAH patients is an unexpected finding.

Not a single risk stratification scale discriminated the actual risk of mortality between the intermediate- and high-risk patients in the entire IPAH population and the elderly group. The REVEAL score exhibited better discrimination of the intermediate- and high-risk groups in IPAH patients with LHD than the REVEAL 2.0 score. The predictive value for REVEAL 2.0 and REVEAL in the entire IPAH population and patients with LHD was in concordance with that of other studies [6,8,16,17,18]. The ESC/ERS 2015 risk stratification scale provided the highest proportion of intermediate-risk patients in all groups and underestimated the number of high-risk patients, which corresponded with other large registry-based studies [16,17]. The largest number of high-risk patients was stratified using the REVEAL 2.0 scale in the younger and elderly cohorts, as well as in the entire IPAH population in our study. This might be related to the significant contribution of the presence of chronic kidney disease [6,19,20,21] and the severely impaired mobility in elderly patients with comorbidities [16]. However, the decreased glomerular filtration rate is not specific to PAH but it is strongly associated with age, arterial hypertension, and the presence of diabetes mellitus in the general population. Chronic kidney disease, NTproBNP ≥ 1400 pg/mL, and the RV/LV ratio appeared to independently predict death in the entire population of patients in our study. Indeed, NT-proBNP demonstrates high correlations with the major clinical, echocardiography, and hemodynamic determinants of PAH severity [22] and exhibits high predictive value in serial evaluations for long-term survival [23]. Elevated NT-proBNP levels seem to overestimate the PAH risk in elderly patients with decreased eGFR [24].

Functional class III-IV (WHO) demonstrated significant association with survival in the univariate regression analysis but lost its value compared with the presence of chronic kidney disease, NTproBNP ≥ 1400 pg/mL, the RV/LV ratio, and pericardial effusion when multivariate regression analysis was applied. This observation is in line with the absence of a significant association between the 6MWT distance and survival in the entire group. A low 6MWT distance and higher FC (WHO) could reflect at least more prevalent comorbidity (systemic hypertension, COPD, obesity, arthritis) and a low physical fitness in the elderly population, and, therefore, overestimate the severity of IPAH [24]. In this case, CPET is suggested to clarify the mechanisms of exercise intolerance [25]. The excellent correlations of cardiopulmonary parameters with invasive hemodynamics make CPET extremely promising and desirable for a non-invasive assessment of PAH severity and prognosis. The absolute values of peak oxygen consumption and peak minute ventilation per unit of carbon dioxide were included in the risk stratification proposed by the ESC/ERS in 2015. However, these parameters have not been widely used due to their low concordance with 6MWT and the interpretation discrepancies between the absolute and predicted values [26]. We observed the comparable absolute values of peak VO_2_, oxygen pulse, and CO_2_ ventilatory equivalent in two groups, while the predicted values of peak VO_2_, aerobic threshold, and VO_2_/HR were significantly lower in younger patients in comparison with patients over 60 years. This observation suggests a greater compromise of the cardiorespiratory system in young patients. The validation of predictable absolute values for age and sex can significantly improve the relevance of cardiorespiratory testing for risk stratification [27,28].

The absence of constant survival predictors, like etiology of PAH, age, gender, and comorbidity, remains the main drawback of the ESC/ERS risk scale. Cardiac index, stroke volume index, and mixed venous oxygen saturation, used in the ESC/ERS score, are closely interrelated with each other and could add points and overlap the effect of other indicators. The REVEAL and REVEAL 2.0 scales use PVR for the assessment of pulmonary vascular disease severity and prognosis. However, patients with IPAH and PVR <5 WU or >32 WU are quite rarely encountered in clinical practice. Moreover, the variability of the right ventricle‘s pulmonary artery uncoupling has been well defined; therefore, PVR might not reflect prognosis in all cases. A low number of patients with a PVR value of <5 WU (n = 11) and a predicted DLCO > 80% (n = 6) could be additional reasons for the increased number of high-risk patients according to the REVEAL 2.0 score in our study [29]. The implementation of cardiac visualization with magnetic resonance imaging (MRI) in the ESC/ERS 2022 scale might significantly improve early diagnosis of the right heart disadaptation and, therefore, facilitate the discrimination between low- and intermediate-risk patients [30]. In our study, the RV/LV ratio was determined as an independent predictor for survival in the entire population. A positive correlation was established between the RV/LV ratio and pulmonary artery pressures, and PVR in previous studies [31,32]. Elderly patients and patients with H2FpEF ≥ 2 are characterized by a smaller RV/LV ratio, possibly contributing to a phenotype of PH associated with HFpEF or vice versa with IPAH in elderly patients.

At present, there is no specific tool for risk assessment in IPAH with comorbidities. The implementation of IPAH phenotype clustering might be a more effective approach in terms of disease course prediction and choosing the number of PAH drugs for initial therapy [5,13]. Four distinct IPAH clusters were determined by R. Badagliacca et al. (2020) in 252 prevalent patients based on age, gender, right heart remodeling on echocardiography, invasive hemodynamics, and CPET [33]. However, this approach did not take into account comorbidity which could unpredictably modify the disease course. M. Hoeper et al. (2020, 2022) proposed an alternative three-cluster approach to IPAH phenotyping with a consideration of age, gender, smoking history, and diffusion capacity for carbon monoxide. The authors particularly focused on lung disease phenotype identification as it exhibits poor treatment response and the worst prognosis compared to group III PH [34]. Bayesian network modeling in the PHORA calculator might be a new promising approach to individual survival calculations using simultaneous assessments and the interrelationship of weighting demography, clinical, imaging, laboratory, invasive hemodynamics, and exercise testing parameters [35,36]. The implementation of certain comorbidities or main indices of comorbidities might strengthen the PHORA risk estimation.

According to the Global Burden of Disease Study, average life expectancy and aging strongly vary with country and reflect many aspects of countries, including the socio-demographic index, which is considerably lower in the Russian Federation than in the USA, Western Europe, and Japan [37]. The official Rosinfostat data provide 71.2 years as the average life expectancy in the Russian Federation from the years 2010 to 2019 [https://rosinfostat.ru/prodolzhitelnost-zhizni/ (URL accessed on 12 February 2024)]. We chose the age of 60 years as the cut-off for aging because the number of comorbidities considerably increases [38]. Age > 60 years was strongly associated with HFpEF in a Caucasian population in Y. Reddy et al.’s study (2018) (OR (95% CI) 6.2, β = 1.82, AUC 0.704, sensitivity 80%, specificity 60%, *p* < 0.0001) [14]. H. Okura et al. (2009) demonstrated that left ventricular diastolic dysfunction was age-dependent even in healthy people [39]. The challenge in the differential diagnosis of IPAH and PH associated with HFpEF in elderly patients with comorbidity remains evident [40,41,42,43]. In our study, the mean age of the IPAH population was 45 years, which was comparable with the first NIH registry [44]. Patients over 60 years represented 25.2% of the entire population compared to 63% of patients over 65 years in the COMPERA registry [9]. The provocative tests with fluid loading or exercise challenge during the RHC were not part of routine clinical practice at the time of working on the majority of the PAH registries [6,9,17,18,45] and conducting our study. At present, methodological approaches that could improve the reliability of hemodynamic data during RHC are well defined in elderly patients with a presence of LHD [46].

We deliberately excluded patients with clinically significant LHD and unreliable PCWP. We attempted to find out how concomitant controlled left heart disease with preserved left ventricle ejection fraction could modify the pulmonary vascular disease course and presentation. For this purpose, we used the uniform countable heart failure with preserved ejection fraction H2FpEF score and assessed the differences in IPAH patients depending on the H2FpEF score. We did not find any differences in PVR, cardiac index, and PWCP between the young and elderly patients, as well as between the groups with H2FpEF ≥ 2 and H2FpEF ≤ 1. PCWP was almost the same for both groups (8.46 ± 4.6 vs. 8.9 ± 4.7 mmHg, *p* = 0.6) and was lower compared with the PCWP obtained from the COMPERA and Swiss registries (10 ± 3 and 12 ± 7 mmHg, respectively) and N. Öcal et al.’s (2022) data [9,47,48,49]. The majority of patients with H2FPEF score ≥ 2 are treated with loop diuretics that could reduce PCWP. In such cases, fluid loading might not be sufficient for PCWP elevation up to 18–20 mmHg [50]. Exercise testing with PCWP registration might be a more relevant physiological provocative test for HFpEF unmasking [51].

Another issue is the PVR cut-off for the pulmonary vascular disease definition in elderly patients [52,53,54]. PVR was similar in the young and elderly IPAH patients in our study (13.8 ± 6.86 and 12.6 ± 5.27 WU, respectively) and is comparable with the PVR value in young typical IPAH patients in the NIH, ASPIRE, REVEAL, and French registers, whereas PVR in the COMPERA and Swiss registries is lower (9.6 ± 5.5; 8.5 ± 5.2 WU, respectively) [9,48,49]. In the Italian PATRIARCA Registry, PVR is 5.56 ± 3.31 WU in patients with PH associated with HFpEF [55], which is much lower in comparison with the IPAH patients with H2FpEF ≥ 2 in our study (12.5 ± 5.2 WU), respectively. Therefore, we could suggest that even elderly patients with H2FpEF ≥ 2 in our study represented IPAH with mild LHD comorbidity. We assume that the presence of a high PVR, a low cardiac output with normal PCWP value, and a significant predominance of the right chambers over the left according to imaging studies, clinical signs of the right heart failure might indicate IPAH in elderly patients with concomitant non-structural LHD with preserved left ventricular ejection fraction (e.g., severe left ventricular hypertrophy, left heart valve disease, cardiomyopathy, and non-revascularized coronary artery disease). The H2FpEF score might be a promising tool for differentiating the PH group II from group I in patients with mild pulmonary vascular disease (PVR ≤ 5 WU).

The cumulative survival rates of 1 to 10 years of follow-up observed in our study are comparable with other registries, as well as the rate of the initial combined-therapy prescription (46%) [56,57,58]. It should be noted that PAH therapy in Russia is limited to endothelin receptor antagonists (ERA) (bosentan, ambrisentan, macitentan), phosphodiesterase type 5 inhibitor (PDEi-5- sildenafil), prostanoids (inhaled iloprost), IP-receptors agonist (selexipag), and soluble guanylate cyclase stimulator (sGC—riociquat). We have to emphasize that parenteral prostanoids are still not available. The high rate of PAH therapy escalation (from 46 to 89%) might partly explain the relatively high survival in the entire cohort.

The most striking result of our study was a similar survival rate in the elderly and young IPAH patients, even with limited use of initial combination PAH therapy in the elderly patients (12%) in comparison with the young patients (63%). These data are in line with the current ESC/ERS guideline 2022, which propose the initial monotherapy for IPAH patients with LHD or lung disease comorbidity despite the baseline risk stratification. No correlations were found between the initial number of PAH drugs and the survival in groups and the entire population. Similar results were obtained by B. Stubbe et al. (2021) in the analysis of monotherapy prescription in atypical IPAH and PAH group I patients from four PAH referral centers in Germany [59]. The comparable survival in the young and elderly patients in our study might be attributed to the absence of patients with severe lung disease, left heart disease, or pathology, extremely diminishing life expectancy, as well as a high rate of PAH therapy escalation in elderly patients (from 12 to 56.6%) during follow-up. The escalation rate in our study is much higher in comparison to that of M. Hoeper et al.’s study (2022), where only 28% of patients with LHD comorbidities were allocated to the combined PAH therapy on follow-up [17,18]. Even though treatment goals were not met in the majority of cases in the elderly population of IPAH patients with comorbidities in the COMPERA registry and AMBITION study [17,18,60], treatment escalation might improve outcomes in some cases.

No statistically significant difference in survival among patients with different H2FpEF score groups was found. These results may greatly depend on the small number of patients with a high H2pEF score in our study. A. Kianzad et al. (2022) revealed that IPAH patients with certain hemodynamic profiles with a low cardiac index, low PCWP, and high mean PAP exhibited similar responses with PAH therapy even in IPAH patients with a high H2FPEF score in comparison with IPAH patients with a low H2FPEF score [61]. The results of our study confirm the feasibility of the initial monotherapy in elderly IPAH patients with comorbidities. Nevertheless, the presence of the hemodynamic and heart remodeling features of a high PAH risk could be a justification for sequential combination therapy. Unfavorable PAH phenotype with early onset of the pulmonary vascular disease and fast progression despite the number of PAH drugs used might be a reason for unmet expectations regarding the observed survival rate in young IPAH patients [59,60].

Taking into account the number of reasons for pulmonary vasculature and heart remodeling with aging [52,53,62], IPAH identification remains an exciting diagnostic dilemma. A sophisticated diagnostic workup with hemodynamic testing, weighting of hemodynamic parameters and clinical presentation, comorbidity, right and left heart remodeling, and laboratory markers are warranted in the elderly population for true IPAH diagnosis [61,63]. Large-scale, long-term observational studies are needed to achieve personalized IPAH description and a PAH strategy choice in IPAH patients using a multimodal approach and genetic studies [63,64,65].

## 5. Limitations

The presented IPAH cohort might not reflect routine registry practice, as patients with severe comorbidities were not included in the analyses.

The limited number of IPAH patients over 60 years in our registry may reflect poor awareness among general practitioners of IPAH diagnosis in the elderly.

Saline loading and exercise testing during the RHC performance were not performed at the time of conducting the study due to the absence of ESC/ERS 2015 recommendations.

The number of patients with an H2FpEF score ≥ 2 was relatively small; therefore, we could not draw a reliable conclusion regarding the effect of H2FpEF on survival and treatment effect.

## 6. Conclusions

Risk stratification in elderly IPAH patients requires a fundamentally different approach than that of young patients, taking into account the initial limitations in physical performance and comorbidities that interfere with current assessment parameters.

The REVEAL stratification scale reliably stratifies patients at any age with left heart disease comorbidities.

Even mild left heart disease and COPD have a negative impact on exercise tolerance in IPAH patients. We still need to address how to best incorporate aging comorbidities, including left heart disease, mobility, and CKD, and differentiate them from higher-risk PAH features.

The clinical relevance of using the H2FpEF score for elderly patients with precapillary hemodynamic profiles requires further validation in a large cohort of patients.

The initial monotherapy seems to be reasonable in patients over 60 years. Selection tools for initial combination therapy in elderly IPAH patients with comorbidities need to be improved based on prospective observational studies.

## Figures and Tables

**Table 1:** Characteristics of the IPAH patients.

Parameters, n (%); m ± SD; M, IQR; 25;75.	Entire Cohort, n = 119	<60 Years, n = 89 (74.8%)	≥60 Years, n = 30 (25.2%)	*p* Value
Age (years)	45.19 ± 16.18	37.8 ± 11	67.4 ± 5.2	<0.0001
Male, n (%)	22 (18.48)	18 (20.2)	4 (13.3)	0.4
BMI (kg/m^2^)	26.5 ± 6.1	25.47 ± 6.7	29.1 ± 5.7	0.79
Smoking, n (%)	25 (21)	18 (20.2)	7 (23.3)	0.7
Comorbidity				
Hypertension, n (%)	49 (41.17)	23 (25.8)	26 (86.6)	<0.001
Ischemic heart disease, n (%)	13 (10.9)	6 (6.7)	7 (23.3)	0.01
COPD, n (%)	13 (10.9)	6 (6.7)	7 (23.3)	0.01
Chronic kidney disease, n (%)	10 (8.4)	5 (5.6)	5 (16.6)	0.059
Diabetes mellitus, n (%)	16 (13.4)	9 (10.1)	7 (23.3)	0.06
Symptoms				
Syncope, n (%)	43 (36.1)	33(37.1)	10 (33.3)	0.7
Edema ***, n (%)	67 (56)	49 (55)	18 (60)	0.63
Atrial fibrillation/atrial flutter, n (%)	18 (15.1)	11 (12.3)	7 (23.3)	0.12
Chest pain, n (%)	42 (35.3)	32 (35.9)	10 (33.3)	0.79
Functional class (WHO)				
FC I, n (%)	2 (1.68)	2 (2.25)	0 (0.0)	0.4
FC II, n (%)	25 (21.0)	22 (24.7)	3 (10)	0.08
FC III, n (%)	75 (63.0)	56 (62.9)	19 (63.3)	0.96
FC IV, n (%)	17 (14.3)	9 (10.1)	8 (26.6)	0.027
Pulmonary function test, n	102	72	30	
TLC, %	95.9 ± 16.27	97.2 ± 16.27	91.37 ± 15.8	0.9
FEV1, %	93.3 ± 16.5	94.5 ± 14.8	89.1 ± 21	0.16
DLCO, %	59.1 ± 15.7	60.2 ± 16.2	55.5 ± 13.7	0.25
Exercise test				
Patients with 6MWT, n	118	89	29	
6MWT, m	317.9 ± 130.9	343 ± 126.8	240.7 ± 113.4	<0.001
SatO_2_ baseline, %	94.0 ± 4.87	94.3 ± 4.9	93.4 ± 4.5	0.6
Patients with CPET, n	76	62	12	
VO_2_ peak, mL/kg/min	13.7 ± 4.3	14.0 ± 4.6	12.2 ± 2.9	0.23
VO_2_ peak Predicted, %	55.7 ± 19.2	53.4 ± 19.3	67.5 ± 14.2	0.02
VO_2_/kg AT, mL/kg/min	13.1 ± 3.2	13.2 ± 4.1	11.8 ± 3.3	0.3
VO_2_/kg AT Predicted, mL/kg/min	51.3 ± 18.4	48.7 ± 17.6	68.8 ± 14.8	0.003
VO_2_/HR	6.97 ± 2.4	6.96 ± 2.5	7.04 ± 1.5	0.92
VO_2_/HR Predicted, %	65 ± 19.8	62.6 ± 19.7	77.6 ± 15.1	0.01
Ve/VCO_2_ peak	50.4 ± 14.1	50.4 ± 14.5	50.37 ± 12.7	0.95
Ve/VCO_2_ AT	47 ± 14.6	45.7 ± 13.1	56.5 ± 21.3	0.049
Breathing reserve, %	50.45 ± 14	52.37 ± 13.5	40.58 ± 13.2	0.007
Laboratory parameters				
Uric acid, µmol/L	458.5 ± 157.2	451.9 ± 157.4	478.9 ± 158.9	0.5
Bilirubin, µmol/L	12 (20; 29)	12 (21; 29)	12 (15; 28)	0.32 *
Hemoglobin, g/L	146.2 ± 21.3	146.1 ± 22.5	146.4 ± 17.4	0.8
Creatinin, µmol/L	80.5 (71; 93)	80 (80; 91.5)	84.5 (71; 97)	0.14 *
eGFR, mL/min/1.73 m^2^	78.9 ± 23.9	83.6 ± 24.1	65.2 ± 17.5	<0.001
NT-proBNP, pg/mL	1050 (341; 2063)	1443 (482; 307)	895 (295; 2050)	0.29 *

* Mann–Whitney U test; *** Fisher exact two-tailed test applied to differences between young group and elderly group of patients; AT, anaerobic threshold; BMI, body mass index; COPD, chronic obstructive pulmonary disease; CPET, cardiopulmonary exercise test; DLCO, diffusion capacity of the lungs for carbon monoxide; FC, functional class; FEV1, forced expiratory volume in one second; F, female; eGFR, estimated glomerular filtration rate; H2FpEF-score (Heavy-Hypertensive (2 or more antihypertensive medicines)—paroxysmal or persistent Atrial Fibrillation—Elder (age > 60 years)—Filling pressure (Doppler echocardiographic E/e(l) ratio > 9)); IQR, interquartile range; 6MWT, 6 min walk test; NT-proBNP, N-terminal pro-brain-type natriuretic peptide; VO_2_/HR, oxygen pulse; VO_2_/HR Predicted, oxygen pulse predicted; PAH, pulmonary arterial hypertension; TLC, total lung capacity; VO_2_ peak, peak oxygen consumption; VO_2_ peak Predicted, predicted peak oxygen consumption; VO_2_/kgAT, peak oxygen consumption at anaerobic threshold; VE/VCO_2_, minute ventilation per unit carbon dioxide production.

**Table 2:** Echocardiography data in young group and in elderly group of IPAH patients.

Parameters, n (%); m ± SD; M, IQR; 25; 75.	Entire Cohort, n = 119	<60 Years, n = 89 (74.8%)	≥60 Years, n = 30 (25.2%)	*p* Value
LAVI, mL/m^2^	25 (20.5; 30.0)	25 (20.0; 29.0)	29 (24.0; 36.0)	0.002 *
LVEDD, mm	39.4 ± 6.1	38.7 ± 6.2	41.4 ± 5.2	0.036
SV, mL	44.2 ± 14.6	43.2 ± 14.4	47.3 ± 15.3	0.18
EF LV, %	64.5 ± 7.1	64.5 ± 7.2	64.3 ± 6.9	0.88
IMMLV, g/m^2^	70.2 ± 26.7	64.5 ± 24.2	87.8 ± 26.8	0.0001
Ve/Va ratio	0.89 (0.67; 1.27)	1.0 (0.69; 1,32)	0.72 (0.64; 0.82)	0.004 *
Lateral E/e’ ratio	6.39 (5.0; 9.05)	6.05 (4.5; 7.8)	9.05 (6.6; 10.0)	0.001 *
RA area, mm^2^	26.05 (21.0; 7.7)	26 (21.0; 38.0)	27.0 (24.0; 33.0)	0.83 *
RVEDD, mm	47.7 ± 7.7	47.9 ± 7.8	46.9 ± 7.5	0.53
RV free wall, mm	6.8 ± 2.1	6.83 ± 1.9	6.69 ± 2.46	0.76
TAPSE, mm	15.58 ± 4.2	15.49 ± 4.14	15.9 ± 4.4	0.69
RV/LV ratio	1.25 ± 0.3	1.28 ± 0.35	1.15 ± 0.24	0.06
ePASP, mmHg	94.2 ± 24.4	94.2 ± 24.6	94.1 ± 24.4	0.98
Pericardial effusion, n (%)	28 (23.5)	20 (22.5)	8 (26.6)	0.6

* Mann–Whitney U test; ePASP—estimated pulmonary artery systolic pressure; LAVI—left atrium volume index; LVEDD, left ventricular end-diastolic diameter; SV—stroke volume; LVEF—left ventricular ejection fraction; IMMLV—left ventricular myocardial mass index; Ve/Va ratio—the ratio of the early (E) to late (A) ventricular filling velocities; lateral E/e’ratio—the ratio of early diastolic mitral inflow velocity to early diastolic lateral mitral annulus velocity; ePASP—estimated pulmonary arterial systolic pressure; RA area—right atrium area; RVEDD—right ventricular (RV) end-diastolic diameter in the apical 4-chamber view; TAPSE, tricuspid annular plane systolic excursion; RV/LV ratio, RVEDD to LVEDD ratio (measured in the 4-chamber apical view).

**Table 3:** Invasive hemodynamics in young group and elderly group of patients with IPAH.

Parameters, n (%); m ± SD	Entire Cohort, n = 119	<60 Years, n = 89 (74.8%)	≥60 Years, n = 30 (25.2%)	*p* Value
HR, beat/min	83 ±13.9	85.7 ± 13.58	74.9 ± 11.9	<0.001
SBP, mmHg	122.6 ± 21.7	116.9 ± 18.4	139.3 ± 22.4	<0.0001
DBP, mmHg	74.2 ± 12.2	73.3 ± 12.4	76.9 ± 11.2	0.16
mBP, mm Hg	89.4 ± 15	86.3 ± 14.3	98.5 ± 13.6	<0.0001
SPAP, mm Hg	88.9 ± 21.6	90.2 ± 23.2	85 ± 15.9	0.26
DPAP, mm Hg	35.4 ± 11.8	37.2 ± 12.4	30.5 ± 8.2	0.007
mPAP, mm Hg	55.4 ± 13.4	56.8 ± 14	51.4 ± 13.4	0.055
RAP, mm Hg	8.47 ± 5.67	8.16 ± 5.6	9.36 ± 5.8	0.32
PCWP, mm Hg	8.8 ± 4.7	8.9 ± 4.7	8.46 ± 4.6	0.6
CI, L/min/m^2^	2.14 ± 0.68	2.16 ± 0.73	2.1 ± 0.49	0.6
PVR, dyn/s/sm^−5^	1112.1 ± 550.7	1143.6 ± 587.2	1018.5 ± 419.3	0.28
PVR, Wood units	13.8 ± 6.86	14.26 ± 7.3	12.6 ± 5.27	0.27
SvO_2_, %	61.5 ± 8.98	61.5 ± 9.5	61.6 ± 7.2	0.94

HR, heart rate; SBP, systolic blood pressure; DBP, diastolic blood pressure; mBP, mean arterial pressure; SPAP, systolic pulmonary arterial pressure; DPAP, diastolic pulmonary arterial pressure; mPAP, mean pulmonary artery pressure; RAP, right atrial pressure; PCWP, pulmonary capillary wedge pressure; CI, cardiac index; PVR, pulmonary vascular resistance; SvO_2_, mixed venous oxygen saturation.

**Table 4:** Risk stratification, PAH therapy, and survival in IPAH patients.

Parameters, n (%);	Entire Cohort, n = 119	<60 Years, n = 89	≥60 Years, n = 30	*p* Value
ESC criteria (2015)				
Low risk, n (%)	14 (11.76)	11 (12.3)	3 (10)	1 **
Intermediate risk, n (%)	88 (73.1)	64 (71.9)	24 (80)	0.4 **
High risk, n (%)	17 (14.3)	14 (15.7)	3 (10)	0.5 **
REVEAL				0.2
Low risk, n (%)	47 (39.5)	39 (43.8)	8 (26.6)	0.13 **
Intermediate risk, n (%)	44 (36.9)	31 (34.8)	13 (43.3)	0.5 **
High risk, n (%)	28 (23.5)	19 (21.3)	9 (30)	0.3 **
REVEAL 2.0				0.03
Low risk, n (%)	38 (32)	31 (34.8)	7 (23.3)	0.26 **
Intermediate risk, n (%)	25 (21)	22 (24.7)	3 (10)	0.12 **
High risk, n (%)	56 (47)	36 (40.4)	20 (66.6)	0.02 **
Death, n (%)	42 (35.3)	30 (33.7)	12 (40)	0.65 **
Initial PAH therapy 0–6 months from IPAH diagnosis				
Monotherapy	71 (59.6)	48 (53.9)	23 (76.6)	0.03
Dual therapy	47 (39.4)	40 (44.9)	7 (23.3)	0.05
Triple therapy	1 (0.84)	1 (1.1)	0 (0)	-
PAH therapy at the time of analysis				
Monotherapy	30 (25.2)	17 (19.1)	13 (43.3)	0.006 **
Dual therapy	57 (47.8)	43 (48.3)	14 (46.6)	0.8 **
Triple therapy	32 (26.9)	29 (32.6)	3 (10)	0.017 **

* Mann–Whitney U test; ** Fisher exact two-tailed test applied to differences between young group and elderly group of patients.

**Table 5:** Exercise performance, laboratory, hemodynamic data, and PAH therapy in the entire IPAH cohort with different H2FpEF scores.

Parameters, n (%); m ± SD; M, IQR; 25;75.	IPAH with H_2_FPEF-Score (≤1) *n* = 74 (62.2%)	IPAH with H_2_FPEF-Score (≥2) *n* = 45 (37.8%)	*p* Value
III–IV FC, n (%)	53 (71.6)	39 (86.6)	0.07
Death, n (%)	24 (32.4)	18 (40)	0.4
Double PAH therapy at baseline, %	32 (43)	16 (35.5)	0.4
Double PAH therapy at time of analyses, n (%)	37 (50)	20 (44.4)	0.5
Triple PAH therapy at time of analyses, n (%)	23 (31)	9 (20)	0.2
Loop diuretics, (%)	41 (55.4)	38 (84.4)	0.0005
6MWT, m	348 ± 130	269 ± 117	0.001
DLCO, %	59.7 ± 16	58.3 ± 15	0.7
eGFR, mL/min/1.73 m^2^	85.4 ± 24	68.4 ± 19.6	0.0001
NT-proBNP, pg/mL	781 (327; 1968)	1363 (530; 2307)	0.6
RV/LV ratio	1.28 ± 0.36	1.18 ± 0.26	0.09
HR, beats/min.	85.4 ± 14.2	78.9 ± 12.6	0.01
mBP, mm Hg	86.1 ± 13.9	94.8 ± 15.2	0.0016
mPAP, mm Hg	55.9 ± 13.5	54.6 ± 13.3	0.6
RAP, mm Hg	7.4 ± 5.2	10.2 ± 6.0	0.01
PCWP, mm Hg	8.5 ± 4.8	9.4 ± 4.6	0.3
CI, L/min/m^2^	2.2 ± 0.7	2.1 ± 0.5	0.3
PVR, WU	14.6 ± 7.6	12.5 ± 5.2	0.1
SatO_2_, %	94.9 ± 3.8	93.2 ± 3.6	0.02
SvO_2_, %	62.5 ± 9.4	59.9 ± 8.1	0.2

CI, cardiac index; DLCO, diffusion capacity of the lungs for carbon monoxide; FC, functional class; eGFR, estimated glomerular filtration rate; H2FpEF-score (heavy-hypertensive (2 or more antihypertensive medicines)—paroxysmal or persistent atrial fibrillation—elder (age > 60 years)—filling pressure (Doppler echocardiographic E/e(l) ratio > 9)); 6MWT, 6 min walk test; NT-proBNP, N-terminal pro-brain-type natriuretic peptide; HR, heart rate; mBP, mean arterial pressure; mPAP, mean pulmonary artery pressure; RAP, right atrial pressure; PCWP, pulmonary capillary wedge pressure; CI, cardiac index; PVR, pulmonary vascular resistance; RV/LV ratio, right ventricular end diastolic dimension to left ventricular end diastolic dimension ratio; SatO_2_, arterial blood oxygen saturation; SvO_2_, mixed venous oxygen saturation.

**Table 6:** Risk factors for survival (All-cause Mortality) using univariate Cox regression analysis in the entire cohort of IPAH patients (n = 119) [HR (95% CI; *p*-value)].

Parameters	Beta	St.Error	Beta 95% Lower	Beta 95% Upper	t-Value	Wald Statist.	*p*	Risk Ratio	Risk Ratio 95% Lower	Risk Ratio 95% Upper
CKD	1.157174	0.445595	0.283825	2.030523	2.596921	6.743999	0.00941	3.180930	1.328200	7.618071
FC PAH	0,861336	0,273444	0,325396	1,397275	3.149958	9.922233	0.001634	2.366319	1.384579	4.044164
6MWT, m	−0.003073	0.001195	−0.005415	−0.000732	−2.57265	6.618517	0.010097	0.996931	0.994600	0.999268
RAP, mmHg	0.079858	0.025287	0.030297	0.129419	3.158084	9.973494	0.001590	1.083133	1.030760	1.138167
mPAP, mmHg	0.029847	0.012368	0.005606	0.054089	2.413192	5.823494	0.015819	1.030297	1.005621	1.055579
CI<2.0, l/min/m^2^	1.244547	0.351667	0.555293	1.933801	3.538994	12.52448	0.000402	3.471361	1.742451	6.915749
PVR, WU	0.0940	0.024646	0.045712	0.14232	3.8146	14.5518	0.00001	1.09857	1.046773	1.152950
SvO2,%	1.254724	0.338465	0.591345	1.918102	3.707104	13.74262	0.000210	3.506869	1.806417	6.808027
DLCO,%	−0,030248	0.011386	−0.052564	−0.007933	−2.65671	7.058087	0.007895	0.970205	0.948794	0.992098
RV/LV ratio	1.498785	0.459166	0.598826	2.398724	3.264123	10.65450	0.001099	4.476202	1.819981	11.00912
Effusion	0.877420	0.334028	0.222737	1.532102	2.626786	6.900007	0.008624	2.404687	1.249492	4.627896
NTproBNP > 1400 pg/ml	1.467548	0.375716	0.731158	2.203938	3.906005	15.25687	0.000094	4.338583	2.077486	9.060620
Uric acid, µmol/l	0.003130	0.001057	0.001058	0.005202	2.960681	8.765630	0.003072	1.003135	1.001058	1.005216
Creatinine, µmol/l	0.013037	0.003677	0.005830	0.020243	3.545718	12.57211	0.000392	1.013122	1.005847	1.020449

CI, confidential interval; CI, cardiac index; CKD, chronic kidney disease; DLCO, diffusion capacity of the lungs for carbon monoxide; HR, hazard ratio; FC, functional class; PVR, pulmonary vascular resistance; RV:LV ratio, RVEDD to LVEDD ratio (measured in the 4-chamber apical view); mPAP, mean pulmonary artery pressure; 6MWT, 6 minute walk test; NT-proBNP, N-terminal pro-brain-type natriuretic peptide; SVO_2_, mixed venous oxygen saturation; RAP, right atrial pressure.

**Table 7:** Risk factors for death (all-cause mortality) using multivariate Cox regression analysis in the entire cohort of IPAH patients [HR (95% CI; *p*-value)].

Parameters	HR	95% HR Lower CL	95% HR Upper CL	*p* Value
CKD	0.235222	0.075860	0.72937	0.012192
FC III–IV	0.477839	0.155053	1.47259	0.198445
RV/LV ratio	5.974738	1.727930	20.65911	0.004743
Pericardial effusion	1.194996	0.512635	2.78564	0.679938
NT-proBNP > 1400 pg/mL	3.188396	1.341593	7.57746	0.008657

CI, confidence interval; CKD, chronic kidney disease; HR, hazard ratio; FC, functional class; RV/LV ratio, RVEDD to LVEDD ratio (measured in the four-chamber apical view); NTproBNP, N-terminal pro-brain-type natriuretic peptide.

## Data Availability

The data that support the findings of this study are available from the corresponding author upon reasonable request.

## Supplementary Materials

The following supporting information can be downloaded at: https://www.mdpi.com/article/10.3390/life14020259/s1.
