# Supplementary material for: Elderly Patients with Idiopathic Pulmonary Hypertension: Clinical Characteristics, Survival, and Risk Stratification in a Single-Center Prospective Registry
Source: Life (Basel). 2024 Feb 16;14(2):259. doi: 10.3390/life14020259 (PMC10890450; doi:10.3390/life14020259)
Supplement: Supplementary file 1 [file life-14-00259-s001.zip › life-2810223-supplementary.pdf]

Supplementary Material

Elderly Patients with Idiopathic Pulmonary Hypertension: Clinical Characteristics, Survival, and Risk Stratification in a Single-Center Prospective Registry

Natalia Goncharova *,†, Kirill Lapshin, Aelita Berezina, Maria Simakova, Alexandr Marichev, Irina Zlobina, Narek Marukyan, Kirill Malikov, Alexandra Aseeva, Vadim Zaitsev and Olga Moiseeva †

Almazov National Medical Research Centre, Ministry of Health of Russia, Saint Petersburg 197341, Russia;

* Correspondence: ns.goncharova@gmail.com

† Current address: Akkuratova Street, 2, Saint-Petersburg 197341, Russia.

*** Information about the area supervised by the reference center**

PH reference center covers the area of the Northwest Region of Russia with Caucasian population. The average population of the Northwest region is 13,799,091 people with an average population density of 8.1/km^2 and with female to male predominance in a ratio of 1.18:1.0. Hospitalization and outpatient visits with the necessary examinations carried out using compulsory health insurance for the residents of Russia. Patients from the rural areas of the Northwest region admitted to the PH referral center through the Telemedicine system. According to the Rosinfostatistics, the average number of doctors was 479 per 100 thousands population. Twelve regional PH centers are engaged in management of PAH patients. Right heart catheterization (RHC) carried out only in inpatient cardiology departments in Russian Federation. All incident PAH patients have to be evaluated in PH referral center with the RHC and some noninvasive studies performance. Chest MSCT with the contrast study, pulmonary function test, abdominal ultrasound, ECG, viral serological tests (e.g., HIV), antinuclear antibody test and standard blood tests could be accepted from regional outpatient clinics. First noninvasive evaluation in PH reference center usually includes echocardiography, 6 minute walk test and cardiorespiratory testing, diffusion capacity of the lungs for carbon monoxide assessment and V/Q lung scan. Patients with IPAH, being residents of the Russian Federation, receive PAH-specific therapy in accordance with the Federal Law on Assistance to Patients with Orphan Diseases at the expense of budgetary funds.

**Death certificate and follow-up information request

The cause of death is coded according to the international ICD-10-PCS — Procedure Coding System. Almazov Center is a Federal research and educational medical institution of the Ministry of Health. The Center is obliged to monitor the medical care quality in regions and have an opportunity to request any medical information of the patient who received treatment or diagnostic procedures at the Center. All patients sign an informed consent to authorize the use of anonymized medical data for scientific and educational purposes at the time of hospitalization

or outpatient care. All patients provide information about authorized persons (usually relatives), who are authorized to receive and transmit information about the patient's health status, as needed. Informed consent is standardized by the Ethical committee of the Center and complies with the legislation of the Russian Federation.

Table 6. Risk factors for survival (All-cause Mortality) using univariate Cox regression analysis in the entire cohort of IPAH patients (n=119) [HR (95% CI; p-value)]

Parameters	Beta	St.Error	Beta 95% lower	Beta 95% upper	t-value	Wald Statist.	p	Risk ratio	Risk ratio 95% lower	Risk ratio 95% upper
CKD	1.157174	0.445595	0.283825	2.030523	2.596921	6.743999	0.00941	3.180930	1.328200	7.618071
FC PAH	0,861336	0,273444	0,325396	1,397275	3.149958	9.922233	0.001634	2.366319	1.384579	4.044164
6MWT, m	-0.003073	0.001195	-0.005415	-0.000732	-2.57265	6.618517	0.010097	0.996931	0.994600	0.999268
RAP, mmHg	0.079858	0.025287	0.030297	0.129419	3.158084	9.973494	0.001590	1.083133	1.030760	1.138167
mPAP, mmHg	0.029847	0.012368	0.005606	0.054089	2.413192	5.823494	0.015819	1.030297	1.005621	1.055579
CI<2.0, l/min/m^2	1.244547	0.351667	0.555293	1.933801	3.538994	12.52448	0.000402	3.471361	1.742451	6.915749
PVR, WU	0.0940	0.024646	0.045712	0.14232	3.8146	14.5518	0.00001	1.09857	1.046773	1.152950
SvO2,%	1.254724	0.338465	0.591345	1.918102	3.707104	13.74262	0.000210	3.506869	1.806417	6.808027
DLCO,%	-0,030248	0.011386	-0.052564	-0.007933	-2.65671	7.058087	0.007895	0.970205	0.948794	0.992098
RV/LV ratio	1.498785	0.459166	0.598826	2.398724	3.264123	10.65450	0.001099	4.476202	1.819981	11.00912
Effusion	0.877420	0.334028	0.222737	1.532102	2.626786	6.900007	0.008624	2.404687	1.249492	4.627896
NTproBNP>1400 pg/ml	1.467548	0.375716	0.731158	2.203938	3.906005	15.25687	0.000094	4.338583	2.077486	9.060620
Uric acid, µmol/l	0.003130	0.001057	0.001058	0.005202	2.960681	8.765630	0.003072	1.003135	1.001058	1.005216
Creatinine, µmol/l	0.013037	0.003677	0.005830	0.020243	3.545718	12.57211	0.000392	1.013122	1.005847	1.020449

CI, confidential interval; CI, cardiac index; CKD, chronic kidney disease; DLCO, diffusion capacity of the lungs for carbon monoxide; HR, hazard ratio; FC, functional class; PVR, pulmonary vascular resistance; RV:LV ratio, RVEDD to LVEDD ratio (measured in the 4-chamber apical view); mPAP, mean pulmonary artery pressure; 6MWT, 6 minute walk test; NT-proBNP, N-terminal pro-brain-type natriuretic peptide; SVO_2, mixed venous oxygen saturation; RAP, right atrial pressure.
